# Intratumoral coagulation by radiofrequency ablation facilitated the laparoscopic resection of giant hepatic hemangioma: a surgical technique report of two cases

**DOI:** 10.18632/oncotarget.18994

**Published:** 2017-07-05

**Authors:** Shaohong Wang, Jun Gao, Mengmeng Yang, Shan Ke, Xuemei Ding, Jian Kong, Li Xu, Wenbing Sun

**Affiliations:** ^1^ Department of Hepatobiliary Surgery, Beijing Chao-Yang Hospital Affiliated to Capital Medical University, Beijing, China

**Keywords:** giant hepatic hemangioma, laparoscopy, resection, radiofrequency ablation

## Abstract

**Background:**

Traditionally, open hepatic resection is the first choice of treatment for symptomatic enlarging hepatic hemangiomas, which requires a large abdominal incision and is associated with substantial recovery time and morbidity. Minimally invasive laparoscopic resection has been used recently in liver surgery for treating selected hepatic hemangiomas. However, laparoscopic liver surgery poses the significant technical challenges and high rate of conversion. Radiofrequency (RF) ablation has been proved feasible in the treatment of hepatic hemangiomas with a size range of 5.0-9.9 cm. It is controversial to treat giant hepatic hemangiomas (≥10.0 cm) by means of RF ablation, due to the low technique success rate and high incidence of ablation-related complications. We aimed to assess the safety and efficacy of combined laparoscopic resection with intratumoral RF-induced coagulation for giant hepatic hemangiomas.

**Methods:**

We treated 2 patients with giant subcapsular hepatic hemangioma (12.0 cm and 13.1 cm in diameters respectively) by laparoscopic resection following intratumoral coagulation of the tumor with RF ablation.

**Results:**

Blood loss during resection was 100 ml (case 1) and 300ml (case 2) respectively. No blood transfusion and dialysis were needed during perioperative period. The two patients were discharged 6 days (case 1) and 12 days (case 2) after surgery without any complications, respectively. Postoperative contrast-enhanced CT follow up showed there was no residual tumor.

**Conclusions:**

It is feasible to treat giant subcapsular hepatic hemangioma by laparoscopic tumor resection boosted by intratumoral coagulation using RF ablation, which may open a new avenue for treating giant hemangioma.

## INTRODUCTION

Hepatic hemangiomas are the most common benign tumors of liver, which are generally asymptomatic and do not need clinical intervention. When hemangiomas are larger than 5.0 cm, cause abdominal symptoms or increase in size during follow-up, radical interventions need to be considered [1, 2].

Open hepatic resection is conventionally the first choice of treatment for symptomatic enlarging hepatic hemangiomas. Despite its well-recognized effectiveness, hepatic resection of hemangiomas usually requires a large abdominal incision and is associated with substantial recovery time and morbidity [3]. In recent years, the well-developed surgical technique of laparoscopic resection has provided great opportunities for treating hepatic hemangiomas in a minimally invasive fashion [4–8]. However, great technical challenges still exist in treating a hemangioma with a size greater than 10.0 cm, which is usually regarded as a relative contraindication due to the lack of sufficient manipulating space and the potential risk of massive intraoperative blood loss [9].

Radiofrequency (RF) ablation has been successfully used in the management of hepatic hemangiomas with a size ranging from 5.0 cm to 9.9 cm [10, 11]. However, the application of RF ablation in treating giant hepatic hemangiomas (≥10.0 cm) remains in debate because of the great technical challenge and high incidence of ablation-related complications. The most severe complication is post ablation renal insufficiency or even renal failure due to an abrupt and massive blood cell lysis [11–13].

RF ablation for hepatic hemangiomas can be performed via percutaneous or laparoscopic approach. The diversity of approaches helps extending the scope of treatment indication. Hepatic hemangiomas deeply located in liver parenchyma are suited to be treated by percutaneous image-guided RF ablation. Subcapsular hepatic hemangioma is suitable to be treated via laparoscopic approach using ultrasound guidance [10–12].

Taking advantage of the prominent thermal coagulative effect of RF ablation on hepatic hemangiomas, we treated 2 patients with subcapsular giant hepatic hemangiomas (12.0 cm and 13.1 cm in diameters respectively) by laparoscopic resection boosted by intratumoral RF-induced coagulation in the past year. The written informed consent was obtained from the patients and the procedure was approved by our institutional investigation and ethics committee of Beijing Chao-Yang Hospital Affiliated to Capital Medical University, according to the standards of the Declaration of Helsinki.

## CASE 1

**Figure 1:**
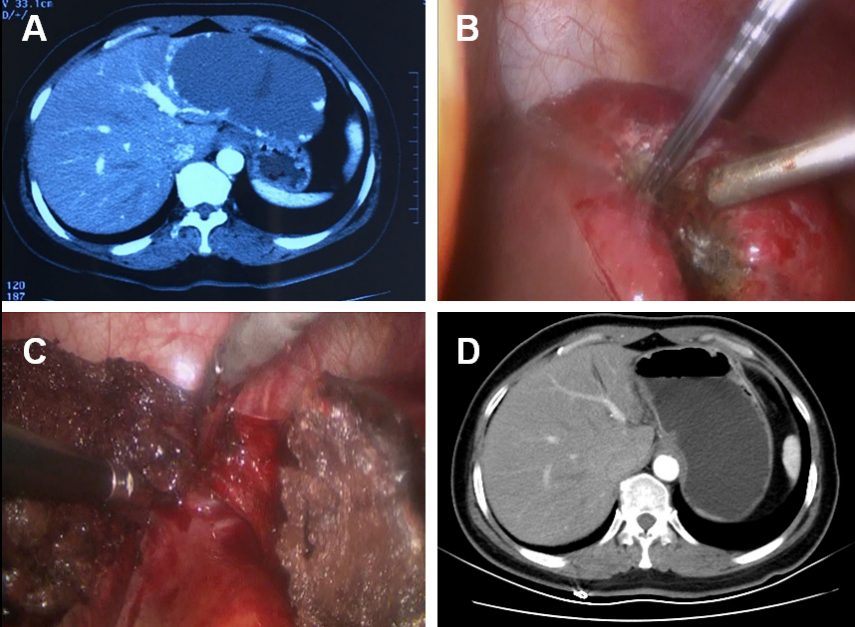
CT and intraoperative photos of case 1 **A**. Contrast-enhanced CT demonstrated a 12.0-cm hepatic hemangioma located in the lateral left lobe of liver. **B**. Intraoperative photo showed the hemangioma at the resection margin shrank significantly after radiofrequency ablation. **C**. The hemangioma was removed and a 1.0-cm band of ablation-coagulated hemangioma was left in place. **D**. Contrast-enhanced CT showed that no residual tissue of the hemangioma exist in the tumor-dissected area.

A 49-year-old female was admitted to our hospital for intermittent onset of vague upper abdominal pain for 3 months, which cannot be attributed to any specific reasons or relived by any medication. Abdominal ultrasonography detected a 12.0 cm mass in the lateral left lobe of the liver. The contrast-enhanced CT confirmed a hepatic hemangioma measuring 12.0 cm ×7.0 cm in diameters (Figure 1A). Upper gastrointestinal endoscopy and colonoscopy ruled out gastrointestinal diseases. Laboratory examinations, including routine blood test, biochemistry test for liver, renal and coagulation function and tumor markers didn't show any abnormal findings. The patient had no history of chronic hepatitis or associated complications.

The choice of using RF ablation to treat such a giant tumor was excluded due to the deemed high incidence of ablation-related complications. A combination therapy of laparoscopic RF ablation and resection was regarded as the optimal choice of treatment. Two hepatobiliary surgeons performed the laparoscopic procedures. Briefly, the patient was placed supine on the surgical table. Under endotracheal anesthesia, a 10.0-mm incision was made at the umbilicus. Following a pneumoperitoneum at 14 mmHg, two additional trocars were placed under direct vision. The laparoscopic exploration found a 12.0-cm hemangioma bulging from the left lateral lobe.

The resection margin was marked by diathermy on the surface of hemangioma about 1.0 cm away from boundary between the normal liver parenchyma and the tumor. RF-induced coagulation was performed along the resection margin by percutaneously introducing RF electrodes under direct visualization. We used internally cooled cluster electrodes, Cool-tip ACTC2025 electrodes and RF generator (Covidien Healthcare, Ireland) for the tumor coagulation. With a 2.5-cm exposed tip, the Cool-tip electrodes can produce ablation zones of 3.0 cm with a single placement of electrodes and a maximum power of 200 W in about 3-5 min. The tissue impedance was continuously monitored on the RF generator monitors throughout the procedure and the power output was adjusted accordingly. A sequential placement of RF electrode into the hemangioma along the resection margin was performed to achieve a complete coagulation of the tumor. A significant shrinking of the hemangioma indicates the complete coagulation of the tumor along the margin (Figure 1B). The hepatic hemangioma was dissected along the coagulation-marked resection margin using harmonic scalpel and a 1.0-cm band of coagulated hemangioma was untouched and left in place (Figure 1C). We removed the dissected tumor tissue using a surgical retrieval bag through the umbilical port and the tissue was analyzed by pathology. The coagulation time was 55 min and the tumor dissection time was 30 min. The total number of punctures was 8. Blood loss during resection was 100 ml. Hepatic cavernous hemangioma was confirmed by histology. The patient was discharged 6 days after surgery without any complications. The abdominal pain disappeared after surgery. Postoperative contrast-enhanced CT follow up showed there was no residual tumor one month after the surgery (Figure 1D). No appearances of late complications have been observed for 14 months since surgery.

## CASE 2

**Figure 2:**
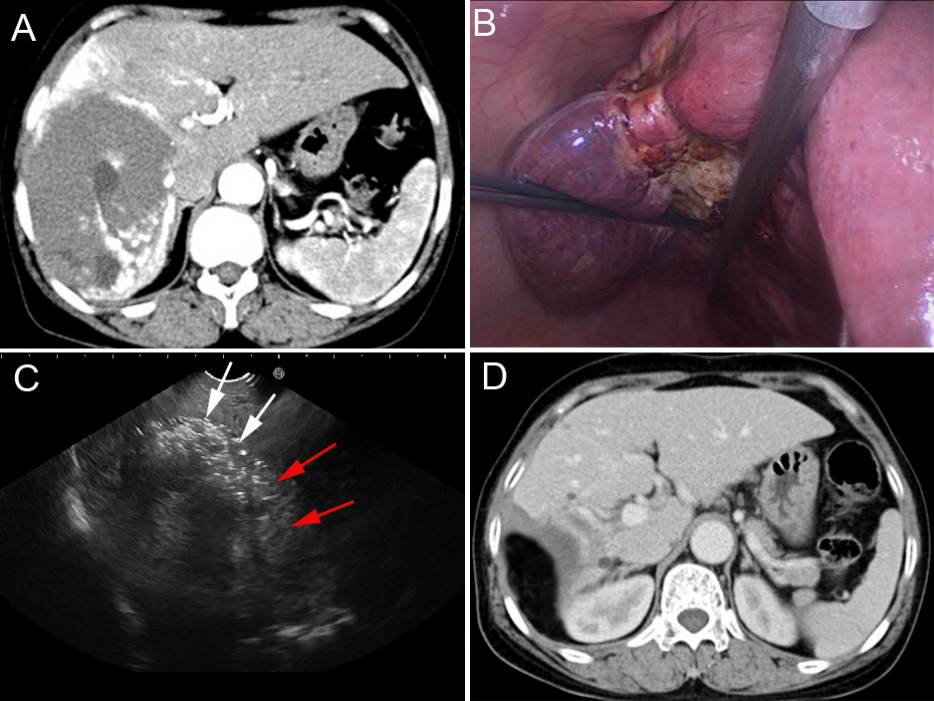
CT and intraoperative photos of case 2 **A**. Contrast-enhanced CT demonstrated a 13.1 cm hepatic hemangioma in the right lobe. **B**. Radiofrequency ablation induced the significant shrinking of the hemangioma along the resection margin. **C**. Intraoperative ultrasound imaging was used to determine the boundary (red arrows) of hepatic hemangioma (white arrows) in liver parenchyma. **D**. Contrast-enhanced CT shows that the hemangioma was completely resected without residual tissue.

A 60-year-old female was admitted to our hospital because of the enlarging subcapsular hepatic hemangioma on regular imaging follow-up within 3 years. No tumor mass was palpable by physical examination. Contrast-enhanced CT showed a typical hepatic hemangioma in the right lobe (13.1 cm×9.3 cm) (Figure 2A). Laboratory test prior to the tumor resection didn't find any abnormal values regarding the liver, renal and coagulator function or tumor markers.

A consensus was reached in the case discussion, attended by a multidisciplinary panel of experts in hepatobiliary tumor treatment, that a combination therapy of RF ablation with laparoscopic resection is the optimal treatment for this patient. Three hepatobiliary surgeons performed the laparoscopic procedures. Briefly, under general anesthesia, the patient was placed supine position on the surgical table. Pneumoperitoneum (CO_2_ at 14 mmHg) was created and the abdomen was explored thoroughly by laparoscope through a 10.0-mm umbilical port. One 12.0-mm subxiphoid port at the midline of abdomen and the other two 5.0-mm right subcostal ports were created. Intraoperative ultrasonography confirmed the CT findings and was used to identify the hepatic veins. Laparoscopic ultrasonography of the liver was performed using a EUP-OL531 laparoscopic ultrasound probe and the HI VISION Preirus ultrasound system (Hitachi Medical Corp., Tokyo, Japan).

The resection margin was marked by diathermy on the surface of hemangioma 1.0 cm away from the border between the normal liver parenchyma and the tumor. RF-induced coagulation was performed along the resection margin by placing the Cool-tip ACTC2025 electrodes into the tumor, which was precisely guided by real-time ultrasound imaging. The substantial coagulation was achieved while the hemangioma tissue along the ablation margin shrank significantly following the RF ablation (Figure 2B). The hepatic hemangioma was dissected along the coagulative necrosis using harmonic scalpel. During the tumor dissection, RF ablation should be repeatedly applied to the tumor if further coagulation is needed. Intraoperative ultrasound imaging was used to determine the boundary of hepatic hemangioma in liver parenchyma, and the ablated lesion became hyperechoic because of outgassing from heated tissues (Figure 2C). The majority of the hemangioma was removed with a 1.0-cm width of coagulated hemangioma left in place. For this patient the coagulation time was 95 min and the dissection time was 50 min. The total number of punctures was 13. Blood loss during resection was 300 ml. In the course of recovery post the procedure, the patient experienced hyperbilirubinemia (42.4 μmol/L of total bilirubin), elevated serum transaminase (801.5 U/L AST and 302.2 U/L ALT) and serum creatinine (190.8 ɥmol/L). All these abnormalities seen in laboratory test resolved after conservative treatment. No blood transfusion and dialysis were needed during perioperative period. A pathological examination confirmed the hepatic cavernous hemangioma. The patient was discharged 12 days after surgery. Three months post the surgery, contrast-enhanced CT confirmed that the hemangioma was completely dissected without any residual tissue (Figure 2D). No appearances of late complications have been observed for 9 months since surgery.

## DISCUSSION

Our results show that laparoscopic resection of hemangioma boosted by intratumoral coagulation by RF ablation was feasibly used to treat giant hepatic subcapsular hemangioma with low loss of blood and negligible complications. The novelty of this technique lies in the fact that a completely coagulated zone created by sequential RF ablation along the dissection margin warranted the successful removal of the tumor tissue without occluding the hepatic vessels before the tumor dissection.

Most incidentally identified and asymptomatic hepatic hemangiomas do not need medical interventions. The indications for treatment of hepatic hemangiomas are the maximum diameter of tumor >5.0 cm; on regular imaging follow up, tumor gaining an enlargement of more than 1.0 cm within 2 years; persistent hemangioma-related abdominal pain or discomfort with the definite exclusion of other gastrointestinal diseases which cause the epigastric pain via gastroscopy examinations [11,12].

Currently the utilization of RF ablation alone to treat giant hepatic hemangiomas larger than 10.0 cm remains in controversial [10–13]. Park et al [10] reported a technical failure rate of 40% by percutaneous RF ablation of hemangiomas ≥ 10.0 cm. Our group [11] also confronted the same technical difficulties while using RF ablation alone to treat 17 hemangiomas with the sizes greater than 10.0 cm, even though we used clustered electrodes in 16 patients. Although a high rate of complete ablation was achieved (82.4%, 14/17), complications post ablation were also seen in all of 16 patients, including significant systemic inflammatory responses (one patient) and acute respiratory distress syndrome (one patient), which may be due to the long ablation time. Therefore, our group [12] designed and implemented a new strategy of RF ablation to treat giant hepatic hemangiomas using (1) cool-tip cluster electrodes and (2) cautiously ablating the tumor by monitoring the conditions of the patients during the ablation procedure. When the patient's body temperature exceeded 39°C or hemoglobinuria occurred, we ended up the procedure and a repeat session was rescheduled. The complete ablation was achieved in 19/21 (90.5%) of the patients and ablation-related complications reduced to 47.6% (10/21). Despite the minor complications, the relatively high complication rate is still unacceptable, which indicates that there are limitations of using RF ablation alone to treat giant hemangiomas.

In recent years, the fast development of minimally invasive liver surgery techniques has enabled the successful laparoscopic resection of selected hepatic hemangiomas [4–8]. However, significant technical difficulties still exist in laparoscopic resection of hepatic hemangiomas with relatively high rate of conversion to open surgery, which has been reported in sporadic publications. The high risk of uncontrollable blood loss is the most important concern in the published reports of laparoscopic liver resection [4–8]. Furthermore, the giant size of hepatic hemangiomas is the most significant risk factor of massive intraoperative blood loss while lobectomy, which usually requires blood transfusion [14]. In order to control the bleeding at the surgical wound in the liver parenchyma, a few measures have been reported, such as the utilization of RF energy to create a desiccation zone prior to hepatic dissection [15–20]. RF-assisted hepatectomy may offer the potential advantages of a bleeding-free procedure, shorter operation time and reduced morbidity. However, the technique has some limitations. The RF ablation cannot be used at the area close to the liver hilum, since heat may damage biliary structures resulting in subsequent bile leakage and/or abscess formation. On the other hand, it cannot control the bleeding from blood vessels measured larger than 4 mm in diameter, such as hepatic or portal vein branches [15–20].

Our technique that combined laparoscopic resection with intratumoral RF-induced coagulation has the advantages in three aspects. Firstly, being different from the RF-assisted liver resection described in the literature, in which the coagulated transaction margin is in the normal liver tissue near the periphery of the tumor [15–20], our study involved the modification of the technique by coagulating the dissection margin in the hemangioma abutting the normal liver parenchyma. Since no vascular biliary bundle was included in the resection plane and no normal liver tissue was ablated, this technique does not cause any damages to biliary structures and hepatic vessels. Secondly, RF-induced coagulation spared the clamping of the hepatic vessels, which ensures the feasible dissection of giant hepatic hemangiomas. The complete ablation of hepatic hemangioma can result in the scarring and collapse of the tumor tissue, which facilitate the resection of the tumor. Thirdly, comparing to treating the tumor with RF ablation alone, the technique involved the ablation of the tumor tissue at the resection margin rather than the total hepatic hemangioma, thus shortened the ablation time and avoided the incidence of severe ablation-related complications. To the best of our knowledge, this is the first study that evaluated the therapy of giant subcapsular hepatic hemangioma by combining intratumoral RF-induced coagulation with laparoscopic tumor resection.

The major limitations of our study include its retrospective nature, only two cases, and the short follow-up period.

In conclusion, our study suggests that it is feasible to treat giant subcapsular hepatic hemangioma by the technique of laparoscopic resection boosted by intratumoral RF-induced coagulation, which may be recommended as the alternative treatment for symptomatic enlarging giant hepatic hemangioma.

This study was supported by the program for high-level technical talents in Beijing health system (2015-3-025) and grant from the National Natural Science Foundation of China (No. 81572957).
